# The effects of ambient temperature on cerebrovascular mortality: an epidemiologic study in four climatic zones in China

**DOI:** 10.1186/1476-069X-13-24

**Published:** 2014-04-01

**Authors:** Yanshen Zhang, Shanshan Li, Xiaochuan Pan, Shilu Tong, Jouni JK Jaakkola, Antonio Gasparrini, Yuming Guo, Sheng Wang

**Affiliations:** 1 Department of Occupational and Environmental Health, School of Public Health, Peking University, Beijing 100191, China; 2 Department of Epidemiology and Biostatistics, School of Population Health, University of Queensland, Brisbane, QLD 4006, Australia; 3 School of Public Health and Institute of Health and Biomedical Innovation, Queensland University of Technology, Brisbane, Australia; 4 Center for Environmental and Respiratory Health Research, Institute of Health Sciences, University of Oulu, Oulu, Finland; 5 Department of Medical Statistics, London School of Hygiene & Tropical Medicine, London, UK; 6 Shanghai Key Laboratory of Meteorology and Health, Shanghai, China

**Keywords:** Cerebrovascular disease, Meta-analysis, Mortality, Temperature, Time series analysis

## Abstract

**Background:**

Little evidence is available about the association between temperature and cerebrovascular mortality in China. This study aims to examine the effects of ambient temperature on cerebrovascular mortality in different climatic zones in China.

**Method:**

We obtained daily data on weather conditions, air pollution and cerebrovascular deaths from five cities (Beijing, Tianjin, Shanghai, Wuhan, and Guangzhou) in China during 2004-2008. We examined city-specific associations between ambient temperature and the cerebrovascular mortality, while adjusting for season, long-term trends, day of the week, relative humidity and air pollution. We examined cold effects using a 1°C decrease in temperature below a city-specific threshold, and hot effects using a 1°C increase in temperature above a city-specific threshold. We used a meta-analysis to summarize the cold and hot effects across the five cities.

**Results:**

Beijing and Tianjin (with low mean temperature) had lower thresholds than Shanghai, Wuhan and Guangzhou (with high mean temperature). In Beijing, Tianjin, Wuhan and Guangzhou cold effects were delayed, while in Shanghai there was no or short induction. Hot effects were acute in all five cities. The cold effects lasted longer than hot effects. The hot effects were followed by mortality displacement. The pooled relative risk associated with a 1°C decrease in temperature below thresholds (cold effect) was 1.037 (95% confidence interval (CI): 1.020, 1.053). The pooled relative risk associated with a 1°C increase in temperature above thresholds (hot effect) was 1.014 (95% CI: 0.979, 1.050).

**Conclusion:**

Cold temperatures are significantly associated with cerebrovascular mortality in China, while hot effect is not significant. People in colder climate cities were sensitive to hot temperatures, while people in warmer climate cities were vulnerable to cold temperature.

## Background

Climate change will be one of the most serious challenge for human health in the 21st century, as it will directly or indirectly affect most populations [1]. Future climate change will increase the frequency, intensity and duration of heat waves [2]. Extreme temperatures have significant impacts on health [3]. For example, over 700 excess deaths were observed during one day of the 1995 Chicago heat wave [4]. There were 15,000 excess deaths in the 2003 France heat waves [5,6] and over 70,000 deaths across Europe [7,8]. There were 274 excess cardiovascular deaths during the 1987 Czech Republic cold spells [9], and 370 excess deaths during the 2006 Moscow cold spells [10].

Exposure to both low and high ambient temperatures increase the risk of death and therefore the temperature-mortality relations appear J-, V- or U-shaped, with thresholds corresponding to the lowest mortality [11-14]. Temperature thresholds for an increased mortality are generally higher in warmer climates [12,15,16], as people adapt to their local climates, through physiological, behavioral and cultural adaptation. So we need to consider human capacity to adapt to varied climates and environments.

Many personal and environmental factors may modify the effects of temperature on human health, including age, gender, presence of chronic diseases, economic, demographic factors, the intensity of urban heat islands, housing characteristics, access to air conditioning and availability of health care services [3]. Therefore it is informative to examine city-specific temperature-mortality associations. Populations in developing countries are anticipated to be especially sensitive to impacts of climate change, as they have limited adaptive capacity and more vulnerable people [1]. To date there has been little research in developing countries [17], including China [11].

Previous studies have reported that the elderly and women are more vulnerable to extreme temperatures than the young and men, respectively [18,19]. People with cardiovascular or respiratory diseases are more susceptible to adverse effects of extreme temperatures than healthy people [17,20,21]. Cerebrovascular death was ranked sixth for the causes of disability adjusted life years worldwide [22]. There is a strong seasonal trend in cerebrovascular death [23,24]. However, few studies have examined the short-term and delayed effects of ambient temperature on cerebrovascular mortality. To our knowledge, no previous study has examined the impacts of temperature on cerebrovascular mortality in cities located in different climatic zones in China.

The primary objective of our study was to assess the associations between short-term ambient temperature exposure and mortality from cerebrovascular disease. The secondary objective was to compare these associations in cold and moderate climatic zones in China and to evaluate whether the temperature thresholds for adverse effects vary between the climatic zones.

## Methods

### Data collection

**Figure 1:**
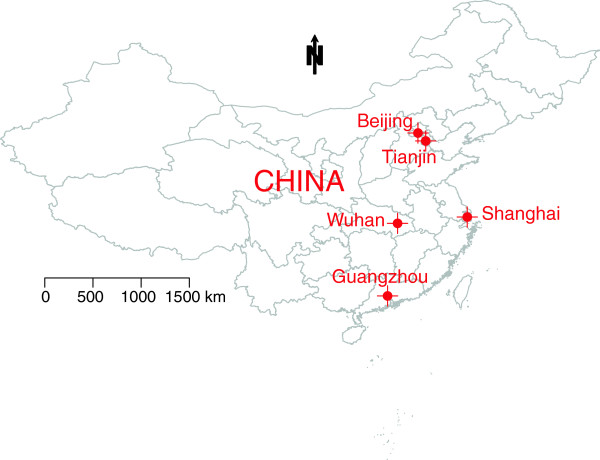
The locations of Beijing, Tianjin, Shanghai, Wuhan and Guangzhou in China.

We obtained data on the daily numbers of deaths from cerebrovascular diseases, weather conditions and average levels of air pollution from five cities (Beijing, Tianjin, Shanghai, Wuhan, and Guangzhou) in China (Figure 1). Beijing and Tianjin are located in northern China, and have clearly four seasons, with cold, windy, dry winters, and hot, humid summers. Shanghai is located in eastern China and has a warm spring, a hot rainy summer, a cool autumn and an overcast cold winter. Wuhan is in central China and is a humid subtropical city, with an oppressively humid summer. Guangzhou is in southern China and has a humid subtropical climate, with long humid and scorching summer, and short winter.

We requested data on the daily counts of death from cerebrovascular diseases from the National Disease Surveillance Points (DSP) System of Chinese Centre for Disease Control and Prevention (China CDC) from January 1, 2004 to December 31, 2008. We used cerebrovascular mortality data from Beijing (Dongcheng district, with 25.34 km^2^ and 626,655 population in 2006), Tianjin (Hongqiao district, with 21.30 km^2^ and 542,264 population in 2006), Shanghai (Luwan district, with 8.05 km^2^ and 371,783 population in 2006), Wuhan (Jiangan district, with 64.24 km^2^ and 706,795 population in 2006) and Guangzhou (Yuexiu district, with 33.82 km^2^ and 419,998 population in 2006). A sentinel surveillances system was used to monitor the mortality data in these five counties. This system was used to confirm and track the causes of death for each person by trained workers. Cerebrovascular deaths were classified according to the International Classification of Diseases, 10th revision (ICD-10: I60–I69).

We requested daily meteorological data on mean temperature and relative humidity from the China Meteorological Data Sharing Service System for each city. We requested daily air pollution data on particulate matter less than 10 μm in aerodynamic diameter (PM_10_), and nitrogen dioxide (NO_2_) from: the Beijing, Tianjin, Shanghai, Wuhan, and Guangzhou Environmental Monitoring Centers.

### Data analysis

We applied a time-series method for statistical analyses. We fitted Poisson regression models to examine the city-specific temperature-mortality relations. We allowed for an over-dispersion in deaths with a quasi-Poisson link function [25]. We assumed the association between temperature and cerebrovascular mortality was non-linear [11]. Mortality risk depends not only on exposure to the current day’s temperature, but also on several previous days’ exposure (lag effects) [26]. We used a natural cubic spline for lag days to overcome the issue that temperatures within a couple of days are strongly correlated [27]. We used a distributed lag non-linear model (DLNM) to smooth temperature and lags simultaneously [27,28]. We used 20 lag days for temperature, as most studies have shown that cold effects can last for weeks, while hot effects are usually acute with some harvesting effects [11-14,29]. We used a natural cubic spline for time to control for seasonal patterns and long-term trends in deaths. We controlled for day of the week as a categorical variable. We controlled for relative humidity, PM_10_, and NO_2_ using natural cubic splines. The following model was used in each city:

$$Y_{t}∼\;\mathrm{Poisson}\left(\mu_{t}\right)$$

(1) $$\begin{array}{l} \mathrm{Log}\;\left(\mu_{t}\right)\;=\;\alpha +\beta T_{t,l}+S\;\left(RH_{t,}3\right)+S\;\left(PM_{10t,}3\right)\\ \;+S\;\left(NO_{2t,}3\right)+S\;\left(\mathrm{time},6\times \mathrm{year}\right)\\ \;+\mathrm{\gamma DO}W_{t}=\alpha +\beta T_{t,l}+\mathrm{COVs} \end{array}$$

where *t* is the day of the observation; *Y*_*t*_ is the number of cerebrovascular deaths on day *t*; α is the intercept; *T*_*t,l*_ is a matrix obtained by the DLNM using five degrees of freedom for temperature and four degrees of freedom for lag days with a natural cubic spline [11], *β* is vector of coefficients for *T*_*t,l*_, and *l* is the lag days. *S(.)* is a natural cubic spline. Three degrees of freedom were used to smooth current day’s relative humidity, PM_10_ and NO_2_ according to previous studies [11,12,14,29]. A natural cubic spline with 6 degrees of freedom per year was used to control for season and long-term trend, as sensitivity tests showed that the estimated temperature effects were then stabilised. DOW_t_ is day of the week on day *t*, and ***γ*** is vector of coefficients.

To capture the relationship between temperature and cerebrovascular mortality over lag days, we plotted the relative risks against temperature and lags. To identify the temperature-mortality thresholds, we plotted the overall effects of temperature on cerebrovascular mortality over lag days.

Our initial analyses found that the temperature-mortality relationships were J-shaped in all five cites, with thresholds for cold and hot effects. We therefore assumed the cold effect was linear below the threshold while the hot effect was linear above threshold, and modelled the lag effects using a natural cubic spline with 4 degrees of freedom. Formula [1] was altered by modifying the β*T*_*i,l*_ term into two linear threshold terms:

(2) $$\mathrm{Log}\left(\mu_{t}\right)=\alpha +\beta_{c}TC_{t,l}+\beta_{H}TH_{t,l}+\mathrm{COVs}\cdot \cdot ,$$

where *TC*_*t,l*_ is a matrix obtained by applying a linear basis (using DLNM) to temperature below the threshold and 4 degrees of freedom natural cubic spline for a 20-day lag. *TH*_*t,l*_ is a matrix obtained by applying a linear basis (using DLNM) to temperature above the threshold and 4 degrees of freedom natural cubic spline for a 20-day lag.

The temperature threshold in model [2] was determined using the Akaike information criterion for quasi-Poisson models (Q-AIC) [28,30]. For example, in Beijing, Temperatures from −5 to 10°C (in 0.1°C gaps) were tested, with the threshold chosen as that which gave the smallest AIC. We estimated the relative risk of cerebrovascular death associated with a 1°C decrease in temperature below the threshold (cold effect) and a 1°C increase above the threshold (hot effect).

We used a random effects meta-analysis to pool cold and hot effects across the five cities, respectively, based on the restricted maximum likelihood [31]. The estimated effects at lags 0–2, 0–13, and 0–20 days were pooled separately, as our initial results showed that hot effects were acute, while cold effects were delayed and lasted more than 7 days.

Sensitivity analyses were performed by modifying the smoothing degrees of freedom and spline type (polynomial, B-spline) for time, temperature, lags, PM_10_, and NO_2_. To check whether 20-lag days were enough to capture the temperature effects on cerebrovascular mortality, we also extended the maximum lag days to 21–42 days. We also performed a sensitivity analysis using the 10th and 90th percentile temperatures as cold and hot thresholds, respectively, and estimated the increased risk of cerebrovascular mortality for a 1°C decrease (increase) in temperature below (above) the cold (hot) threshold.

All statistical tests were two-sided and values of p-values of less than 0.05 were considered statistically significant. The correlations between daily weather conditions and air pollutants were summarised using Spearman’s correlation because some of the pollutants were clearly not normally distributed. The R software (version 2.11.2) was used for all analyses. The “mgcv” was used to perform Poisson regression models. The “dlnm” package was used to fit DLNM [27,28]. The “metafor” package was used for the meta-analysis [32].

## Results

Between 2004 and 2008, there were 2,835 cerebrovascular deaths in Beijing, 4,723 in Tianjin, 2,883 in Shanghai, 6,028 in Wuhan, and 3,839 in Guangzhou. The average daily mean temperature was lowest in Beijing (13.6°C), followed by Tianjin (13.3°C), Shanghai (17.6°C), Wuhan (18.2°C), and Guangzhou (22.9°C) (Table 1). Generally, Beijing Tianjin, and Wuhan had higher PM_10_ concentration than Shanghai and Guangzhou. All cities had similar levels of NO_2_ pollution.

**Table 1:** Summary statistics for daily temperature, relative humidity, air pollutants and cerebrovascular deaths in five Chinese cities during 2004 to 2008

**City**	**Variable**	**Min**	**25%**	**Median**	**75%**	**Max**	**Mean**	**SD**
Beijing	Temperature (°C)	−10.1	3.5	14.8	23.6	32.1	13.6	10.9
	Relative humidity (%)	8	34	52	68	97	52	20
	PM_10_ (μg/m^3^)	10.0	78.3	128.0	184.0	600.0	144.6	91.3
	NO_2_ (μg/m^3^)	14.0	47.1	60.9	76.8	214.4	64.2	25.7
	Deaths	0	1	2	2	8	2	1
Tianjin	Temperature (°C)	−10.5	3.0	14.5	23.4	31.3	13.3	11.1
	Relative humidity (%)	13	46	60	73	97	59	18
	PM_10_ (μg/m^3^)	11.4	72.0	92.0	123.2	755.7	104.2	55.4
	NO_2_ (μg/m^3^)	9.0	30.6	41.2	53.3	146.6	43.8	19.1
	Deaths	0	1	2	4	10	3	2
Shanghai	Temperature (°C)	−3.2	10.0	18.7	25.2	33.8	17.6	9.0
	Relative humidity (%)	31.0	63.0	71.0	79.0	95.0	70.4	11.8
	PM_10_ (μg/m^3^)	12.0	52.0	76.0	114.0	600.0	89.2	53.6
	NO_2_ (μg/m^3^)	12.8	43.2	54.4	72.0	171.2	58.4	22.8
	Deaths	0	1	1	2	8	2	1
Wuhan	Temperature (°C)	−2.7	10.3	19.6	26.1	34.2	18.2	9.4
	Relative humidity (%)	21	61	70	78	97	70	13
	PM_10_ (μg/m^3^)	0.0	80.0	114.0	150.0	350.0	120.3	55.0
	NO_2_ (μg/m^3^)	0.0	36.8	48.0	64.0	153.6	51.6	21.5
	Deaths	0	2	3	4	12	3	2
Guangzhou	Temperature (°C)	5.4	18.5	24.4	27.9	34.2	22.9	6.2
	Relative humidity (%)	20	63	72	80	95	70	13
	PM_10_ (μg/m^3^)	8.0	48.0	73.0	105.1	329.1	81.6	44.4
	NO_2_ (μg/m^3^)	17.1	42.4	58.3	82.5	232.0	66.3	30.4
	Deaths	0	1	2	3	10	2	2

SD: standard deviation.

Daily temperatures were generally positively correlated with relative humidity, except in Wuhan (Table 2). Daily temperatures were negatively correlated with PM_10_ and NO_2_ in all cities except Beijing. PM_10_ was strongly correlated with NO_2_ in all five cities.

**Table 2:** Spearman’s correlations between daily weather and air pollutants in five Chinese cities from 2004 to 2008

**City**	**Variable**	**Temperature**	**Relative humidity**	**PM**_**10**_
Beijing	Relative humidity	0.35**		
	PM_10_	0.00	0.17**	
	NO_2_	−0.17**	0.21**	0.67**
Tianjin	Relative humidity	0.30**		
	PM_10_	−0.07*	0.06*	
	NO_2_	−0.36**	0.08*	0.59**
Shanghai	Relative humidity	0.15**		
	PM_10_	−0.18**	−0.26**	
	NO_2_	−0.39**	−0.16**	0.73**
Wuhan	Relative humidity	−0.09*		
	PM_10_	−0.20**	−0.17**	
	NO_2_	−0.35**	−0.17**	0.76**
Guangzhou	Relative humidity	0.22**		
	PM_10_	−0.08*	−0.15**	
	NO_2_	−0.20**	−0.11**	0.88**

*P < 0.05.

**P < 0.01.

**Figure 2:**
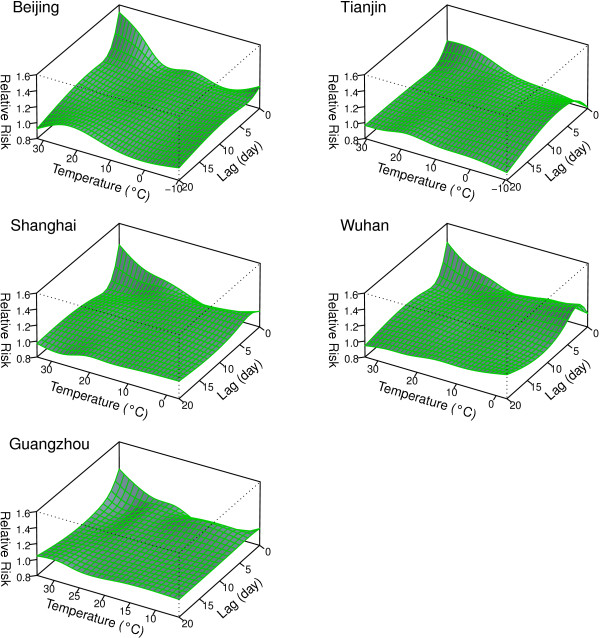
**Relative risks of cerebrovascular disease mortality by temperature (°C) and lag in five Chinese cities during 2004 to 2008.** The risks used 5 degrees of freedom for temperature and 4 degrees of freedom for lag up to 20 days.

Figure 2 shows the relative risk surfaces between temperature and cerebrovascular mortality across all temperatures and lags. The estimated effects of temperature on cerebrovascular mortality were non-linear in all five cities, with higher relative risks at hot and cold temperatures. An increased risk of death for high temperatures appeared quickly in all five cities; the timing of the increased risk of death in cold temperatures were more variable.

**Figure 3:**
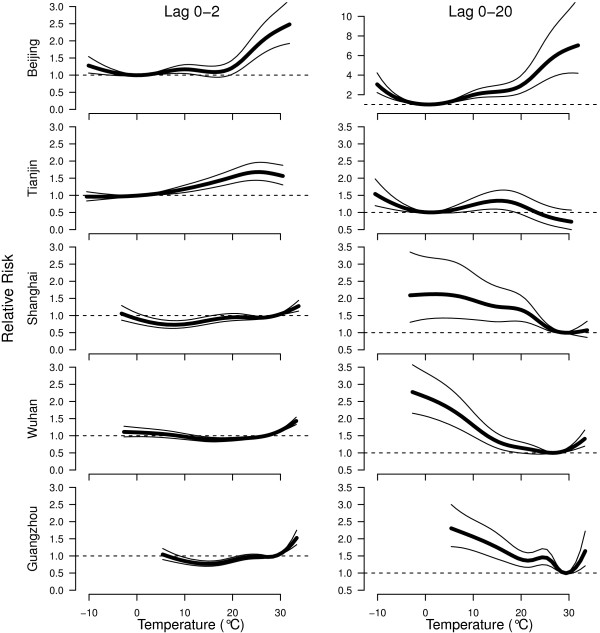
**The estimated overall effects of temperature (°C) over lag 0–2 and lag 0–20 days on cerebrovascular mortality in five Chinese cities.** The thick lines are – point estimate for relative risk, and the thin lines are 95% confidence intervals.

Figure 3 shows the estimated relative risks of cerebrovascular mortality according to temperature over lag 0–2 and lag 0–20 days in the five cities. Generally, there were non-linear relationships between temperature and cerebrovascular mortality in all the cities, with a threshold below (above) which the cold (hot) effect is linear. The thresholds for lag 0–20 days chosen by Q-AIC were: 0.7°C in Beijing, −0.6°C Tianjin, 26.9°C Shanghai, 25°C Wuhan and 27.8°C Guangzhou.

**Figure 4:**
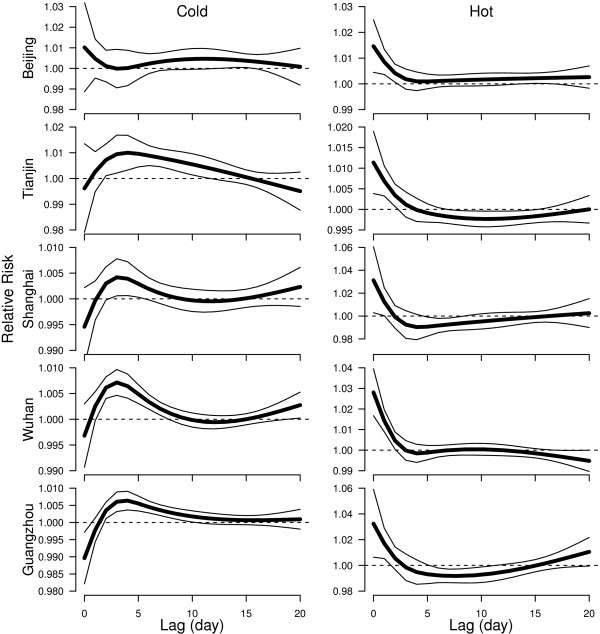
**The effects of a 1°C decrease in temperature below the thresholds (left) and of a 1°C increase in temperature above the thresholds (right) on cerebrovascular deaths over 20 lag days using 4 degrees of freedom.** The thick lines are point estimate for relative risk, and thin lines are 95% confidence intervals. The thresholds were 0.7°C in Beijing, −0.6°C Tianjin, 26.9°C Shanghai, 25°C Wuhan and 27.8°C Guangzhou.

The effects of low and high temperature on cerebrovascular mortality over 20 lag days are shown in Figure 4. The cold effects appeared with a delay in Beijing, Tianjin, Wuhan, and Guangzhou, while in Shanghai the effects of cold had a short induction period. The induction period of the effects of high temperature was short in all five cities. The short-term effects of high temperature were followed by mortality displacement (harvesting) in Tianjin, Shanghai and Guangzhou.

We estimated the effects of temperature on cerebrovascular mortality for a time lag of 0–2, 0–13 and 0–20 days across the five cities (Table 3). The effect estimates for low temperature over lag 0–20 days was 1.070 (95% confidence interval (CI): 1.002, 1.141) per 1°C decrease below threshold in Beijing, 1.072 (95% CI: 1.013, 1.136) in Tianjin, 1.011 (95% CI: 0.966, 1.058) in Shanghai, 1.037 (95% CI: 1.014, 1.061) in Wuhan, and 1.033 (95% CI: 1.003, 1.063) in Guangzhou. The effect estimates for high temperature over lag 0–20 days was 1.062 (95% CI: 1.017, 1.109) per 1°C increase above threshold in Beijing, 0.996 (95% CI: 0.925, 1.073) in Tianjin, 0.972 (95% CI: 0.929, 1.017) in Shanghai, 1.023 (95% CI: 0.978, 1.071) in Wuhan, and 1.014 (95% CI: 0.915, 1.123) in Guangzhou.

**Table 3:** The cumulative cold and hot effects of temperature on cerebrovascular disease death along the lag days, using 4 degrees of freedom natural cubic spline for lag

**Effect type**	**Lag**	**Relative risk (threshold)**				
		**Beijing (0.7°C)**	**Tianjin (−0.6°C)**	**Shanghai (26.9°C)**	**Wuhan (25°C)**	**Guangzhou (27.8°C)**
Cold effect^*a*^	Lag 0–2	1.016 (0.973,1.062)	1.006 (0.970,1.043)	1.023 (0.996,1.050)	1.005 (0.992,1.019)	0.991 (0.975,1.007)
	Lag 0–13	1.050 (0.994,1.110)	1.085 (1.033,1.140)*	1.009 (0.971,1.049)	1.029 (1.011,1.048)*	1.027 (1.004,1.052)*
	Lag 0–20	1.070 (1.002,1.141)*	1.072 (1.013,1.136)*	1.011 (0.966,1.058)	1.037 (1.014,1.061)*	1.033 (1.003,1.063)*
Hot effect^*b*^	Lag 0–2	1.027 (1.005,1.051)*	1.049 (1.011,1.087)*	1.025 (1.004,1.046)*	1.047 (1.022,1.073)*	1.056 (1.000,1.114)
	Lag 0–13	1.044 (1.009,1.081)*	1.012 (0.955,1.072)	0.983 (0.950,1.018)	1.045 (1.006,1.085)*	0.986 (0.909,1.070)
	Lag 0–20	1.062 (1.017,1.109)*	0.996 (0.925,1.073)	0.972 (0.929,1.017)	1.023 (0.978,1.071)	1.014 (0.915,1.123)

*P < 0.05.

^*a*^The relative risk of cerebrovascular death associated with a 1°C temperature decrease below the threshold.

^*b*^The relative risk of cerebrovascular death associated with a 1°C temperature increase above the threshold.

**Figure 5:**
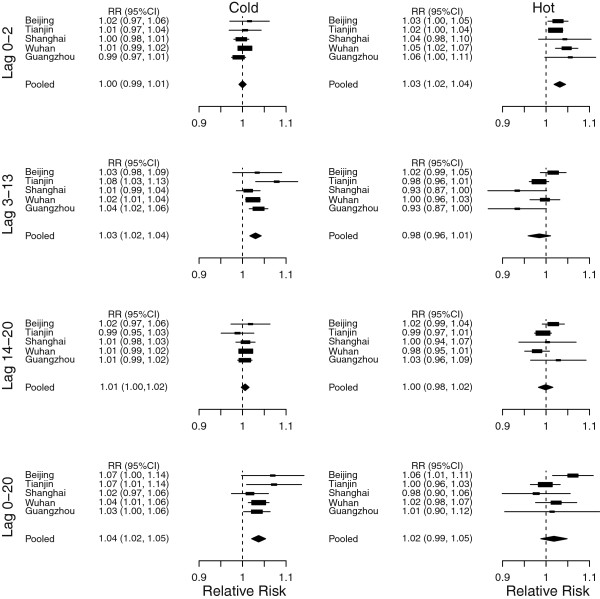
**Meta-analyses for cold effects (left) and hot effects (right) on cerebrovascular deaths at lag 0–2 days (top), lag 0–13 days (middle), and lag 0–20 days (bottom).** The cold effects are relative risks of a 1°C decrease in temperature below the thresholds. The hot effects are relative risks of a 1°C increase in temperature above the thresholds. The thresholds were 0.7°C in Beijing, −0.6°C Tianjin, 26.9°C Shanghai, 25°C Wuhan and 27.8°C Guangzhou.

The summary-effect estimates for low and high temperature at lag 0–2, 0–13 and 0–20 days respectively are shown in Figure 5. The summary-effect estimate for a 1°C decrease in temperature below the threshold (cold effects) was 1.037 (95% CI: 1.020, 1.053) at lag 0–20 days. The summary-effect estimate for a 1°C increase in temperature above the threshold (hot effects) was 1.014 (95% CI: 0.979, 1.050) at lag 0–20 days.

### Sensitivity analysis

The estimated temperature-effects did not change when using more than 6 degrees of freedom per year for time (7–10 per year). Nor did they change when we used 3, 4, and 6 degrees of freedom for temperature, and changed the spline type (B-spline, polynomial) for temperature and lags. The estimated temperature effects were unchanged for 1, 2, 4, and 5 degrees of freedom for PM_10_, and NO_2_, as well as for different types of spline (B-spline, polynomial). We also fitted a time lag of 21–42 days, which also gave similar results. Hence, it appears that the models used in this study adequately captured the main effects of temperature on cerebrovascular mortality.

We used the 10th and 90th percentile temperatures as cold and hot thresholds, and estimated the increase in mortality with a 1°C decrease (increase) in temperature below (above) the cold (hot) threshold. The summary estimates from this analysis (Additional file 1) were similar to the original estimates (Figure 4).

## Discussion

We compared the relations between ambient temperature and mortality from cerebrovascular diseases in cold, moderate and subtropical climatic zones in China and evaluated whether the temperature thresholds for adverse effects vary between the climatic zones. To our knowledge, similar comparisons of temperature threshold effects have not been published previously. This is also the first study to examine the effects of temperature on cerebrovascular mortality in China.

The relations between temperature and cerebrovascular mortality were non-linear in all five cities. Northern cities (with low mean temperature) had lower temperature thresholds for adverse effects than southern cities (with high mean temperature). The effects of low temperature appeared with a delay in Beijing, Tianjin, Wuhan and Guangzhou, whereas in Shanghai the induction period was short. The effects of high temperature had a short induction period in all five cities, followed by mortality displacement. The effects of low temperature on cerebrovascular deaths lasted longer than the effects of high temperature. The summary-effect estimate from the meta-analysis for low temperature was statistically significant for a time lag 0–20 days, while the corrsponding estimate for high temperature was not signigicant at lag 0–20 days.

Few previous studies have examined the effects of temperature on cerebrovascular deaths. A meta-analysis for nine California counties showed that the current day’s apparent temperature had a negative but non-significant impact on cerebrovascular deaths [33]. From a study conducted in Moscow, Revich and Shaposhnikov (2008) reported the temperature threshold for an increased risk of cerebrovascular deaths was 18°C. A 1°C decrease in temperature (lag 0–6 days) below the threshold was associated with 0.8% increase in cerebrovascular mortality, while a 1°C increase in temperature (lag 0) above the threshold associated with 4.7% increase in cerebrovascular mortality [34]. In Yekaterinburg, a 1°C decrease in temperature was related to 1.2% increase for cerebrovascular mortality in winter [35]. Our findings are consistent with these previous studies, which consistently demostrate that low and high temperatures increase the risk of death from cerebrovascular disease.

The present study shows that the temperature thresholds in northern cities (low mean temperature) are lower than in southern cities (high mean temperature). Similar results were reported previously for temperature effects on non-accidental mortality [16]. The thresholds were 16.5°C in Netherlands and 19°C in London , and 29°C in Taiwan [36]. These results suggest that people living in colder climates are more susceptible to hot weather, while people living in warmer climates are more vulnerable to cold. This could be because people have adapted to their local climates. For example, people living in cities in cold climate are better adapted for cold weather by having heaters and wearing warm clothes, but may not have air conditioning needed during warmer days [3].

We investigated the time lag of both low and high temperature effects over 20 days on cerebrovascular mortality. Both city-specific and meta-analysis results showed that cold effects were delayed except Shanghai, while the effects of high temperature appeared with no or short time lag. The effects of low temperature lasted longer than the effects of high temperature. The effects of high temperature were followed by mortality displacement. Similar patterns of time lag were observed in Tianjin, China for the effects of both low and high temperature on total non-accidental, cardiovascular, cardiopulmonary, respiratory mortality [11]. Also, previous studies reported similar time lags for the effects of temperature on cardiovascular death in United States and European countries [26,37]. These findings indicate that using short time lags cannot completely capture the effects of temperature on cerebrovascular mortality, and therefore longer time lags are required to examine the effects of temperature.

The effect of low temperature had a short induction period in Shanghai, whereas in the other four cities the induction period was longer and the cold effects were delayed. This could be explained by a higher sensitivity to the effects of low temperature in the population in Shanghai. There is evidence that climate and population characteristics may modify the impacts of temperature on human health [38]. Social, economic, educational, demographic, infrastructural factors, intensity of urban heat islands, housing characteristics, access to air conditioning, and availability of health care services can also affect the effects of temperature on human health [3].

There is also evidence that high temperatures may induce a recurrent episode in individuals who have previously experienced a myocardial infarction or stroke [39]. Our results suggest that people with cerebrovascular disease are sensitive to high temperature because their ability to cope with heat is already compromised. Several biological mechanisms could explain these findings. The extra heat load may lead to fatal consequences for people with congestive heart failure [40]. Exposure to high temperatures may cause dehydration, salt depletion, and increased surface blood circulation, which can induce a failure of thermoregulation [41]. High temperatures may also be associated with elevated blood viscosity, cholesterol levels and sweating thresholds [42].

Low temperatures can also have an impact on human health [9,43]. Cold has negative effects on myocardial ischemia, myocardial infarction and sudden deaths [44,45]. Exposure to low temperatures is associated with an increase in blood pressure, blood cholesterol, heart rate, plasma fibrinogen, platelet viscosity and peripheral vasoconstriction [46,47]. Skin cooling may increase systematic vascular resistance, heart rate and blood pressure.

This study has several strengths. This is the first study to examine the effects of temperature on cerebrovascular mortality in China. We examined differences in the effects of temperature effects on cerebrovascular mortality in five large cities located in different climatic zones. We also examined both the induction period and the duration of the effects of low and high temperature on cerebrovascular mortality. We used a meta-analysis to summarize the effects in different cities, which strengthened the evidence on the adverse effects of both low and high temperatures on cerebrovascular mortality. We used mortality data from sentinel surveillances, and therefore the data should be reliable. Our findings may have implications in promoting capacity building for local response to climate change.

Several limitations in this study should also be acknowledged. The data are only from five cities, so it is difficult to generalise our results to rural areas. We used the data on temperature and air pollution from fixed sites rather than individual exposure, which was likely to produce some measurement error in exposure assessment. However, a previous study concludes that time series model using fixed site’s temperature has the same ability as spatiotemporal models using spatial temperatures to estimate temperature-mortality relationships [48]. The influence of ozone was not adjusted for, because data on ozone were unavailable. And we cannot exclude completely the confounding effects from influenza epidemic because of data unavailability.

## Conclusion

Our results provide evidence that both low and high temperatures increase the risk of death from cerebrovascular disease in different climatic conditions. People in colder climate seem to be more sensitive to the effects of high temperature, whereas people in warmer climate seem to be more vulnerable to the effects of low temperature. In Beijing, Tianjin, Wuhan and Guangzhou the effects of low temperature were delayed, while in Shanghai with a subtropical climate, the effects of low temperature appeared with no or short time lag. The effects of high temperature had a short induction period in all climatic conditions represented by the five cities.

## Abbreviations

CI: Confidence interval; ICD: International Classification of Diseases; NO2: Nitrogen dioxide; PM10: Particulate matter with aerodynamic diameters less than 10 μm; Q-AIC: Akaike information criterion for quasi-Poisson.

## Competing interest

The authors declare that they have no competing interests.

## Authors’ contributions

YZ and YG designed the study and directed its implementation, including data analysis, writing the paper. SL, XP, JJ, ST, and AG helped conduct the quality assurance, review and edit the paper. YZ and YG prepare the database and conduct the data quality assurance. SW and YG is the guarantor. All authors read and approved the final manuscript.

## Supplementary Material

Additional file 1: Figure S1 Meta-analyses for relative risks of cerebrovascular mortality associated with cold temperature (left) and hot temperature (right) at lag 0–2 days, lag 3–13 days, lag 14–20 days, and lag 0–20 days in five Chinese cities during 2004 to 2008. The relative risks of cerebrovascular mortality associated with cold temperature use a 1°C decrease in temperature below the cold thresholds (10^th^ percentile of temperature in each city). The relative risks of cerebrovascular mortality associated with hot temperature use a 1°C increase in temperature above the hot thresholds (90^th^ percentile of temperature in each city). Click here for file
