# Supplementary material for: The effects of ambient temperature on cerebrovascular mortality: an epidemiologic study in four climatic zones in China
Source: Environ Health. 2014 Apr 1;13:24. doi: 10.1186/1476-069X-13-24 (PMC4021080; doi:10.1186/1476-069X-13-24)
Supplement: Additional file 1: Figure S1 — Meta-analyses for relative risks of cerebrovascular mortality associated with cold temperature (left) and hot temperature (right) at lag 0–2 days, lag 3–13 days, lag 14–20 days, and lag 0–20 days in five Chinese cities during 2004 to 2008. The relative risks of cerebrovascular mortality associated with cold temperature use a 1°C decrease in temperature below the cold thresholds (10th percentile of temperature in each city). The relative risks of cerebrovascular mortality associated with hot temperature use a 1°C increase in temperature above the hot thresholds (90th percentile of temperature in each city). [file 1476-069X-13-24-S1.docx]

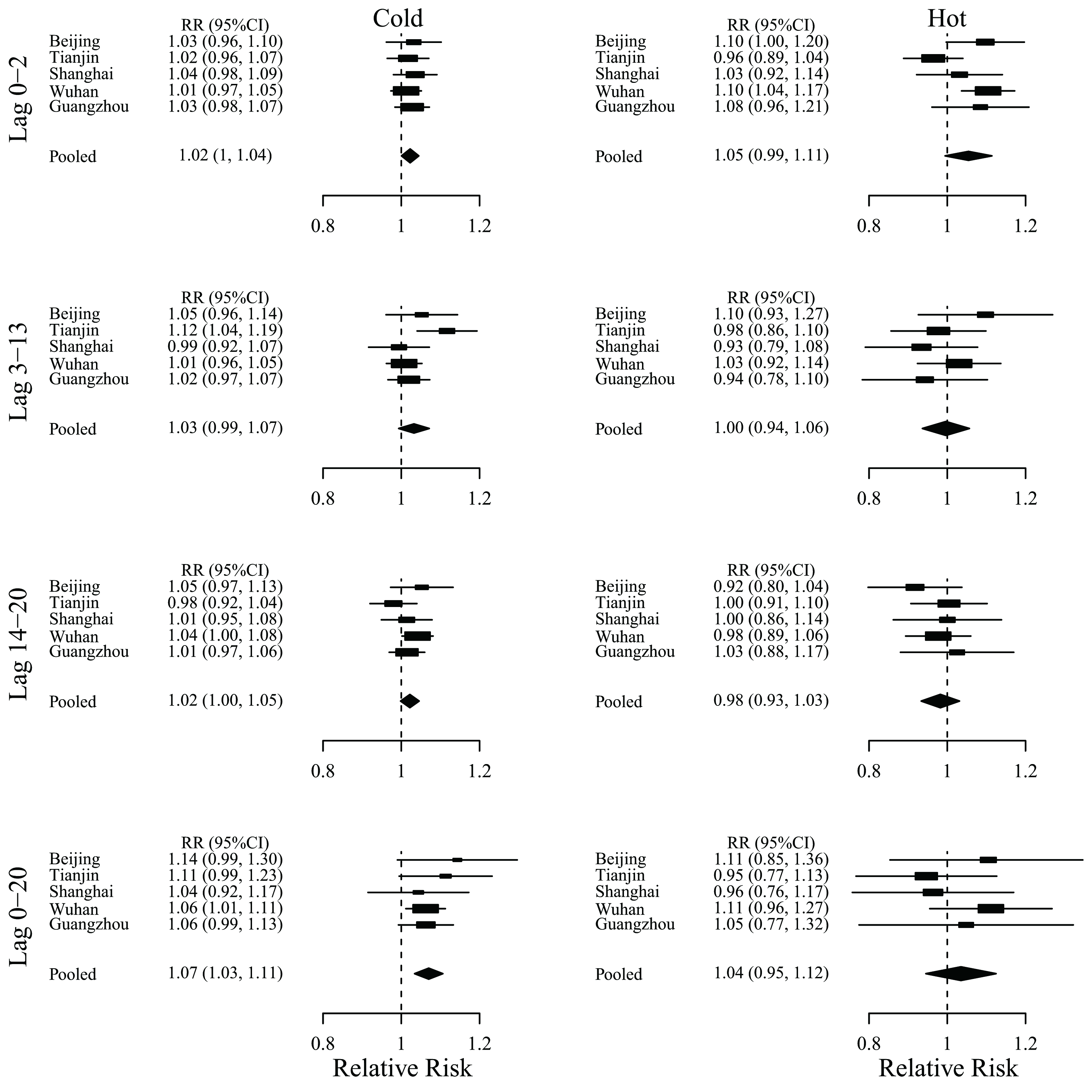


Additional file 1: Figure S1. Meta-analyses for relative risks of cerebrovascular mortality associated with cold temperature (left) and hot temperature (right) at lag 0–2 days, lag 3–13 days, lag 14–20 days, and lag 0–20 days in five Chinese cities during 2004 to 2008. The relative risks of cerebrovascular mortality associated with cold temperature use a 1 °C decrease in temperature below the cold thresholds (10^th^ percentile of temperature in each city). The relative risks of cerebrovascular mortality associated with hot temperature use a 1 °C increase in temperature above the hot thresholds (90^th^ percentile of temperature in each city).
